# The independent and combined effects of aerobic exercise intensity and dose differentially increase post‐exercise cerebral shear stress and blood flow

**DOI:** 10.1113/EP091856

**Published:** 2024-08-14

**Authors:** M. Erin Moir, Adam T. Corkery, Kathleen B. Miller, Andrew G. Pearson, Nicole A. Loggie, Avery A. Apfelbeck, Anna J. Howery, Jill N. Barnes

**Affiliations:** ^1^ Bruno Balke Biodynamics Laboratory, Department of Kinesiology University of Wisconsin‐Madison Madison Wisconsin USA; ^2^ Department of Health and Exercise Science University of St Thomas St Paul Minnesota USA; ^3^ Department of Medicine, School of Medicine and Public Health University of Wisconsin‐Madison Madison Wisconsin USA

**Keywords:** aerobic exercise, blood flow, internal carotid artery, shear stress

## Abstract

This research examined the impact of aerobic exercise intensity and dose on acute post‐exercise cerebral shear stress and blood flow. Fourteen young adults (27 ± 5 years of age, eight females) completed a maximal oxygen uptake (${\dot{V}}_{O_{2}\max}$) treadmill test followed by three randomized study visits: treadmill exercise at 30% of ${\dot{V}}_{O_{2}\max}$ for 30 min, 70% of ${\dot{V}}_{O_{2}\max}$ for 30 min and 70% of ${\dot{V}}_{O_{2}\max}$ for a duration that resulted in caloric expenditure equal to that in the 30% ${\dot{V}}_{O_{2}\max}$ visit (EqEE). A venous blood draw and internal carotid artery (ICA) ultrasound were collected before and immediately following exercise. ICA diameter and blood velocity were determined using automated edge detection software, and blood flow was calculated. Using measures of blood viscosity, shear stress was calculated. Aerobic exercise increased ICA shear stress (time: *P *= 0.005, condition: *P *= 0.012) and the increase was greater following exercise at 70% ${\dot{V}}_{O_{2}\max}$ (∆4.1 ± 3.5 dyn/cm^2^) compared with 30% ${\dot{V}}_{O_{2}\max}$ (∆1.1 ± 1.9 dyn/cm^2^; *P *= 0.041). ICA blood flow remained elevated following exercise (time: *P *= 0.002, condition: *P *= 0.010) with greater increases after 70% ${\dot{V}}_{O_{2}\max}$ (Δ268 ± 150 mL/min) compared with 30% ${\dot{V}}_{O_{2}\max}$ (∆125 ± 149 mL/min; *P *= 0.041) or 70% ${\dot{V}}_{O_{2}\max}$ EqEE (∆127 ± 177 mL/min; *P *= 0.004). Therefore, aerobic exercise resulted in both intensity‐ and dose‐dependent effects on acute post‐exercise ICA blood flow whereby vigorous intensity exercise provoked a larger increase in ICA blood flow compared to light intensity exercise when performed at a higher dose.

## Abstract (highlights)

- **What is the central question of this study?**
  What are the independent and combined effects of exercise intensity and dose on post‐exercise internal carotid artery (ICA) haemodynamics?
- **What is the main finding and its importance?**
  The combined effects of exercise intensity and dose evoked the greatest haemodynamic response whereby vigorous intensity exercise for a longer duration resulted in the greatest increase in post‐exercise ICA blood flow. Therefore, exercise intensity and dose are important considerations for utilizing exercise to improve cerebrovascular function.

## 1 INTRODUCTION

Cerebrovascular dysregulation with age contributes to the development of cognitive decline. In this regard, regular aerobic exercise has demonstrated neuroprotective effects (Barnes, 2015) that may be mediated through improvements in vascular function (Barnes & Corkery, 2018). Following a single bout of aerobic exercise, acute adjustments in peripheral vascular function are apparent including increases in brachial artery flow‐mediated dilatation (Bailey et al., 2017; Johnson et al., 2012; Siasos et al., 2016; Weston et al., 2022). In contrast, alterations in cerebrovascular function following a single bout of aerobic exercise are less clear, with evidence indicating no improvements in cerebrovascular reactivity to steady‐state hypercapnia (Weston et al., 2022) but improved reactivity to transient hypercapnia (Sakamoto et al., 2024). Additionally, following aerobic exercise dynamic cerebral autoregulation was not improved (Willie et al., 2013) and middle cerebral artery blood velocity (MCAv) did not differ from baseline values (Steventon et al., 2018; Weston et al., 2022; Willie et al., 2013). In the peripheral vasculature, shear‐mediated processes facilitate improvements in brachial artery endothelial function following aerobic exercise given the large increases in brachial artery blood flow that occur during exercise (Thijssen et al., 2009). Shear‐mediated mechanisms are also expected to facilitate improvements in cerebrovascular function following aerobic exercise given large increases in internal carotid artery (ICA) blood flow and shear rate (Ogoh et al., 2021; Sakamoto et al., 2024; Sato & Sadamoto, 2010; Sato et al., 2011; Smith et al., 2019) as well as MCAv (Nowak‐Flück et al., 2018; Ogoh et al., 2005; Smith et al., 2019) during exercise. Indeed, a recent investigation demonstrated that an increase in shear rate during exercise was necessary to elicit improvements in post‐exercise cerebrovascular function (Sakamoto et al., 2024). As such, variations in the cerebral shear response during aerobic exercise may explain the incongruent findings in alterations to post‐exercise cerebrovascular function (e.g., cerebrovascular reactivity, cerebral autoregulation) and peripheral vascular function (e.g., flow‐mediated dilatation).

Variations in exercise protocols may differentially impact peripheral and cerebral haemodynamic responses during and following exercise. Specifically, the intensity and dose (i.e., product of exercise intensity, duration, and frequency) of aerobic exercise are two factors that may influence shear responses during and following exercise, which acts as a stimulus for improved vascular function. Indeed, brachial artery blood flow and shear rate were augmented during incremental aerobic exercise (cycling, walking, and kicking exercise) with greater blood flow and shear responses observed with increasing exercise intensity (Thijssen et al., 2009). In contrast to exercise intensity, the overall dose of exercise appeared to have little effect on brachial artery shear rate following treadmill exercise (Johnson & Wallace, 2012). While effects of exercise intensity are observed in the peripheral circulation, evidence in the cerebral circulation is less clear. In the ICA, blood flow differed during cycling exercise performed at varying intensities whereby ICA blood flow was lower during high‐intensity exercise relative to moderate intensity exercise (Sakamoto et al., 2021). In contrast, ICA shear rate was not different during high intensity cycling exercise compared with moderate intensity cycling exercise (Sakamoto et al., 2021). However, ICA blood flow and shear rate were unaltered from pre‐exercise levels in this study and the impact of exercise intensity on blood viscosity may be overlooked with measures of shear rate (Sakamoto et al., 2021). Therefore, the effects of aerobic exercise intensity and dose on cerebral blood flow and shear stress remain unclear.

Additionally, whether the effects of aerobic exercise intensity and dose on cerebral blood flow and shear stress persist beyond exercise remains unknown. Previous investigations have demonstrated that cerebral blood flow and shear stress remain elevated post‐exercise relative to rest (Ogoh et al., 2007, 2021; Sato & Sadamoto, 2010). However, this persisting effect of exercise on cerebral blood flow and shear may be impacted by the intensity and dose of aerobic exercise. Therefore, the primary objective of the present study is to examine the impact of exercise intensity on post‐exercise cerebral shear stress and blood flow measured at the ICA. The secondary objective is to determine the effect of exercise dose on post‐exercise cerebral shear stress and blood flow. This research tested the hypothesis that (1) post‐exercise cerebral shear stress and blood flow would be intensity dependent with greater alterations in ICA haemodynamics following vigorous intensity exercise compared with lower intensity exercise, and (2) post‐exercise cerebral shear stress and blood flow would be influenced by exercise dose whereby a greater dose would elicit greater changes in ICA haemodynamics.

## 2 METHODS

### 2.1 Ethical approval

All study procedures were approved by the University of Wisconsin‐Madison Institutional Review Board (2018‐0783) and performed in accordance with the *Declaration of Helsinki*, with the exception of registration in a database. All participants provided written informed consent prior to study procedures.

### 2.2 Participants

Fourteen healthy young adults (six males, eight females, 20–33 years of age) participated in the present study. Participants were habitual exercisers with a history of running at least three times per week for a minimum of 30 continuous minutes and with experience running on a treadmill (at least 1 year). Participants were free from hepatic disease, renal disease, haematological disease, cardiovascular disease including hypertension, stroke/neurovascular disease and diabetes. Further, participants were not taking blood pressure medications at the time of the study, had a body mass index (BMI) ≤30 kg/m^2^, and did not have a history of depression or other mood‐related disorders. Participants were White (*n* = 11, 92%) and Asian (*n* = 1, 8%).

### 2.3 Laboratory procedures

Participants completed five visits to the laboratory that included a screen visit, familiarization visit, and three study visits. For the screening procedures, participants arrived at the laboratory having fasted for 4 h and having abstained from exercise for 20 h. Participants completed self‐report questionnaires regarding health history and physical activity history, and height and weight were measured. Based on participants’ self‐report physical activity history, the Godin–Shephard Leisure‐Time Physical Activity score and metabolic equivalent of task‐minutes (MET‐min) were determined. Participants underwent carotid artery ultrasonography screening and performed a maximal oxygen uptake (${\dot{V}}_{O_{2}\max}$) test with a modified Balke treadmill protocol. Participants began running at a pace corresponding to 70–80% of their estimated maximal heart rate and the grade increased by 1% every minute until the test was terminated. To be considered a successful ${\dot{V}}_{O_{2}\max}$ test, two of the following three criteria needed to be met: failure to increase ${\dot{V}}_{O_{2}}$ with increasing workload, a respiratory exchange ratio greater than 1.10, or a maximum heart rate within 10 beats/min of age‐predicted maximum. Eight participants met at least two criteria for a successful ${\dot{V}}_{O_{2}\max}$ test while four participants terminated the test due to exhaustion prior to meeting two criteria. Throughout the test, participants were equipped with a 12‐lead electrocardiogram (ECG) and a mouthpiece connected to a metabolic measurement system for metabolic data (TrueOne 2400, ParvoMedics, Salt Lake City, UT, USA).

For the familiarization procedures, participants arrived at the laboratory having fasted for 4 h and abstained from caffeine, chocolate, alcohol, and exercise for 20 h prior to the visit. To familiarize participants with the exercise intensities to be performed on the study days, participants walked and ran on the treadmill. The speed and grade of the treadmill were adjusted to determine the proper workloads to elicit 30% and 70% of the participants’ ${\dot{V}}_{O_{2}\max}$. During the familiarization visit, heart rate was monitored with a telemetric heart rate monitor (Polar H1, Polar Electro Oy, Kempele, Finland) and participants were instrumented with a mouthpiece and nose clip for measures of metabolic data.

Participants completed three study visits in the laboratory: walking at 30% ${\dot{V}}_{O_{2}\max}$ for 30 min (30% ${\dot{V}}_{O_{2}\max}$), running at 70% ${\dot{V}}_{O_{2}\max}$ for 30 min (70% ${\dot{V}}_{O_{2}\max}$), and running at 70% ${\dot{V}}_{O_{2}\max}$ for a duration that resulted in energy expenditure equal to that in the 30% ${\dot{V}}_{O_{2}\max}$ visit (70% ${\dot{V}}_{O_{2}\max}$ EqEE). Energy expenditure was measured via indirect calorimetry (TrueOne 2400). For the 70% ${\dot{V}}_{O_{2}\max}$ EqEE visit, exercise was terminated upon reaching an energy expenditure (kcal) equivalent to that achieved in the 30% ${\dot{V}}_{O_{2}\max}$ visit. The study visits were completed at least 24 h apart (19 ± 15 days between visits). The randomization process allowed the 30% ${\dot{V}}_{O_{2}\max}$ and 70% ${\dot{V}}_{O_{2}\max}$ visits to be randomized for all participants. If the 30% ${\dot{V}}_{O_{2}\max}$ visit was randomized first, the 70% ${\dot{V}}_{O_{2}\max}$ and 70% ${\dot{V}}_{O_{2}\max}$ EqEE visits were also randomized. Because of the energy expenditure matching, the 30% ${\dot{V}}_{O_{2}\max}$ visit had to occur before the 70% ${\dot{V}}_{O_{2}\max}$ EqEE visit. Ahead of each study visit, participants fasted for 4 h, abstained from caffeine, chocolate, alcohol and exercise for 20 h, avoided over‐the‐counter medications, supplements, or non‐steroidal anti‐inflammatory drugs for 5 days prior, and were instructed to maintain a consistent diet and exercise routine for 3 days ahead of each study visit. Female participants were tested for each study visit during days 2–6 of their menstrual cycle. All female participants (*n* = 8) were taking contraceptives (oral: *n* = 7, implant: *n* = 1) during study participation. Participants’ height and weight were measured at each study visit.

Prior to performing the exercise, participants were resting in the supine position and a blood draw via venipuncture was performed. Participants were instrumented with an ECG for measures of heart rate (HR; Cardiocap 5, Datex‐Ohmeda, Madison, WI, USA), finger photoplethysmography for measures of beat‐to‐beat blood pressure, stroke volume and cardiac output (Finapres Nova, Finapres Medical Systems, Amsterdam, The Netherlands), and a nasal cannula for measures of end tidal carbon dioxide ($ET_{CO_{2}}$; Cardiocap 5). The common carotid artery (CCA) and ICA were imaged with duplex Doppler ultrasonography (11L probe, 4.5–12 MHz, GE LOGIQ S8, GE Healthcare, Waukesha, WI, USA). For the exercise protocol, participants began with a 5‐min warm‐up. During the exercise, participants walked or ran at the speed and grade determined during the familiarization visit to correspond with 30% and 70% of their ${\dot{V}}_{O_{2}\max}$. Participants were instrumented with a mouthpiece and nose clip and ${\dot{V}}_{O_{2}}$ was measured throughout the exercise protocol. The speed and grade of the treadmill were adjusted if participants were not within 10% of the desired absolute ${\dot{V}}_{O_{2}}$. Throughout the test, HR was monitored with a telemetric heart rate monitor. During exercise, a finger prick was performed within the last 5 min of exercise for measures of blood lactate (Lactate Plus, Nova Biomedical, Waltham, MA, USA). Immediately following the completion of the exercise protocol, a blood draw via venipuncture was performed and carotid imaging was initiated within 4 ± 1 min with scan time lasting 2.3 ± 1.1 min for the CCA and ICA. Measures of HR, beat‐by‐beat blood pressure and $ET_{CO_{2}}$ were also collected post‐exercise during or immediately following carotid imaging.

### 2.4 Data analysis

Of the 14 participants in the study, two were excluded from analysis for not completing all three study visits due to Covid‐19 laboratory closures. Therefore, data from 12 healthy young adults (five males, seven females, 20–33 years of age) were included in the analysis. Images of the ICA were not obtained in one participant and as such, ICA analysis was completed in 11 participants. Ultrasound images of the CCA (*n* = 12) and ICA (*n* = 11) were analysed offline (Vascular Research Tools, Medical Imaging Applications, LLC, Coralville, IA, USA) for measures of vessel diameter (medial–adventitial border to medial–adventitial border). Additionally, the ultrasound images were analysed for CCA and ICA blood velocity spectrum envelope, which was converted to time‐averaged mean blood velocity (Vascular Research Tools). The time‐averaged mean blood velocity was defined as the mean blood velocity (Ogoh et al., 2021; Sakamoto et al., 2023; Sato & Sadamoto, 2010; Sato et al., 2011). Cross‐sectional area (CSA) was calculated as π × (mean diameter/2)^2^. Blood flow in the CCA and ICA was calculated using the following equation: Blood flow (mL/min) = mean blood velocity × CSA × 60. Blood viscosity increases following aerobic exercise (Connes et al., 2012). As such, whole blood viscosity was measured immediately following blood collection at 6, 12, 30, 60 and 100 revolutions/min and the average viscosity was determined (DV2T Viscometer, Brookfield Ametek, Brookfield Engineering Laboratories, Stoughton, MA, USA). In three participants, blood draws were not obtained post‐exercise during one or more study visits and nine participants with complete blood viscosity data were included in the analysis. Using measures of blood viscosity, shear stress was calculated for the CCA and ICA as (4 × blood viscosity × mean blood velocity)/mean diameter. Shear rate was also calculated as 4 × mean blood velocity/mean diameter for all participants. For measures of HR, stroke volume (SV), cardiac output (CO), systolic blood pressure (SBP), diastolic blood pressure (DBP), mean arterial pressure (MAP) and pulse pressure (PP), an average was taken over the duration of the CCA scans and ICA scans (2.3 ± 1.1 min). Measures of $ET_{CO_{2}}$ were averaged over 5 min. The change in variables (∆) was calculated as the difference between post‐exercise and pre‐exercise values.

### 2.5 Statistical analysis

Statistical analysis was conducted using IBM SPSS Statistics (V28, IBM Corp., Armonk, NY, USA). Statistical significance was set as *P *< 0.05 and all data are presented as means ± standard deviations (SD). Two members of the study team performed analysis of ICA diameters for all three study visits pre‐ and post‐exercise. Interrater reliability analysis was conducted using a two‐way mixed effects model (single measure) and absolute agreement was assessed (ICC = 0.7, *P *< 0.01). Absolute agreement of baseline measures across the three study visits was determined using intraclass correlation (two‐way mixed effects model). A one‐way repeated measures analysis of variance compared blood lactate concentrations across conditions (30% ${\dot{V}}_{O_{2}\max}$, 70% ${\dot{V}}_{O_{2}\max}$, 70% ${\dot{V}}_{O_{2}\max}$ EqEE). Two‐way repeated measures analysis of variance compared all haemodynamic measures between time (pre‐ vs. post‐exercise) and condition (30% ${\dot{V}}_{O_{2}\max}$, 70% ${\dot{V}}_{O_{2}\max}$, 70% ${\dot{V}}_{O_{2}\max}$ EqEE). Significant main effects or significant interactions were assessed using Tukey's *post hoc* analysis. Cohen's effect sizes were calculated with G*Power 3.1.9.6 as *d_z_ *= (mean_1_ – mean_2_)/SD_1_ – SD_2_) (Faul et al., 2007).

## 3 RESULTS

Participant characteristics for the 12 participants with complete data are presented in Table 1. The average exercise intensity attained during the three study visits was 30 ± 1% ${\dot{V}}_{O_{2}\max}$ for the 30% ${\dot{V}}_{O_{2}\max}$ condition, 67 ± 2% ${\dot{V}}_{O_{2}\max}$ for the 70% ${\dot{V}}_{O_{2}\max}$ condition, and 67 ± 2% ${\dot{V}}_{O_{2}\max}$ for the 70% ${\dot{V}}_{O_{2}\max}$ EqEE condition. The length of exercise completed in the 70% ${\dot{V}}_{O_{2}\max}$ EqEE was 13.6 ± 0.7 min. Blood lactate concentrations were 2.0 ± 1.0 mmol during the 30% ${\dot{V}}_{O_{2}\max}$ exercise, 4.1 ± 1.8 mmol during the 70% ${\dot{V}}_{O_{2}\max}$ exercise, and 2.9 ± 0.9 mmol during the 70% ${\dot{V}}_{O_{2}\max}$ EqEE exercise (condition: *P *= 0.006). Blood lactate concentrations during exercise differed between 30% ${\dot{V}}_{O_{2}\max}$ exercise and 70% ${\dot{V}}_{O_{2}\max}$ exercise (*P *= 0.014, *d *= 1.3). In contrast, blood lactate concentrations during exercise did not differ between 70% ${\dot{V}}_{O_{2}\max}$ exercise and 70 ${\dot{V}}_{O_{2}\max}$ EqEE exercise (*P *= 0.157, *d *= 0.7) or between 30% ${\dot{V}}_{O_{2}\max}$ exercise and 70% ${\dot{V}}_{O_{2}\max}$ EqEE exercise (*P *= 0.093, *d *= 0.8).

**TABLE 1:** Participant characteristics and resting haemodynamics.

*n* (females/males)	12, 7/5
Age (years)	27 ± 5
Height (cm)	173 ± 11
Weight (kg)	69 ± 9
BMI (kg/m^2^)	23 ± 2
SBP (mmHg)	117 ± 8
DBP (mmHg)	71 ± 6
MAP (mmHg)	86 ± 6
PP (mmHg)	46 ± 6
HR (bpm)	53 ± 7
Godin score (au)	66 ± 22
MET‐min (min/week)	3693 ± 836
${\dot{V}}_{O_{2}\max}$ (mL/kg/min)	53 ± 9

*Note*: Values are presented as means ± SD. Abbreviations: BMI, body mass index; DBP, diastolic blood pressure; HR, heart rate; MAP, mean arterial pressure; MET‐min, metabolic equivalent of task‐minutes; PP, pulse pressure; SBP, systolic blood pressure; ${\dot{V}}_{O_{2}\max}$, maximal oxygen uptake.

Blood viscosity was determined pre‐ and post‐exercise on all visits in nine participants (Table 2). There was a strong agreement between pre‐exercise blood viscosity measures across conditions (ICC = 0.8, *P *< 0.001). Blood viscosity increased following 70% ${\dot{V}}_{O_{2}\max}$ EqEE (Δ 0.3 ± 0.5 centipoise (cP)) which differed from 30% ${\dot{V}}_{O_{2}\max}$ (Δ −0.2 ± 0.5 cP; *P *= 0.025, *d *= 0.9) but did not differ from 70% ${\dot{V}}_{O_{2}\max}$ (Δ 0.1 ± 0.4 cP; *P *= 0.124, *d *= 0.6). Also, the increase in blood viscosity following 70% ${\dot{V}}_{O_{2}\max}$ exercise did not differ from 30% ${\dot{V}}_{O_{2}\max}$ exercise (*P *= 0.077, *d *= 0.7). Hemodynamics before and following exercise are reported in Table 2. Immediately following exercise, MAP was not different from pre‐exercise values (time: *P *= 0.410, condition: *P *= 0.667, interaction: *P *= 0.245). HR remained elevated following exercise, but the magnitude of the effect differed by condition (time: *P *< 0.001, condition: *P *< 0.001, interaction: *P *< 0.001). The increase in HR following exercise at 70% ${\dot{V}}_{O_{2}\max}$ (Δ 21 ± 9 bpm; *P *< 0.001, *d = *2.3) and the 70% ${\dot{V}}_{O_{2}\max}$ EqEE condition (Δ 18 ± 6 bpm; *P *< 0.001, *d *= 2.7) was greater than the increase following exercise at 30% ${\dot{V}}_{O_{2}\max}$ (Δ 3 ± 5 bpm).

**TABLE 2:** Cardiorespiratory, blood viscosity, and carotid artery parameters before and immediately following exercise.

	30% ${\dot{V}}_{O_{2}\max}$		70% ${\dot{V}}_{O_{2}\max}$		70% ${\dot{V}}_{O_{2}\max}$ EqEE		2‐Way RM ANOVA *P*‐value		
	Pre‐exercise	Post‐exercise	Pre‐exercise	Post‐exercise	Pre‐exercise	Post‐exercise	Time	Condition	Interaction
HR (bpm)	54 ± 8	58 ± 6*	58 ± 9	79 ± 6*	53 ± 7	71 ± 10*	<0.001	<0.001	<0.001
SV (mL)	91 ± 21	96 ± 15	96 ± 14	81 ± 12*	91 ± 14	87 ± 8	0.094	0.142	0.002
CO (L/min)	4.9 ± 1.0	5.2 ± 1.1	5.4 ± 0.9	5.9 ± 1.6	4.7 ± 0.5	6.1 ± 1.0*	0.009	0.092	0.122
SBP (mmHg)	119 ± 16	117 ± 16	123 ± 13	110 ± 11*	117 ± 15	118 ± 13	0.131	0.866	0.024
DBP (mmHg)	67 ± 10	65 ± 10	69 ± 6	67 ± 8	68 ± 9	70 ± 7	0.790	0.279	0.393
MAP (mmHg)	85 ± 11	82 ± 12	87 ± 8	82 ± 8	84 ± 11	86 ± 9	0.410	0.667	0.245
$ET_{CO_{2}}$ (mmHg)	42.4 ± 1.3	40.4 ± 2.3*	40.8 ± 3.4	39.1 ± 2.0	41.2 ± 2.8	39.7 ± 2.5*	<0.001	0.119	0.653
Blood viscosity (cP)	4.1 ± 0.2	3.9 ± 0.4	4.2 ± 0.4	4.3 ± 0.4	4.1 ± 0.5	4.4 ± 0.3	0.512	0.043	0.022
CCA diameter (mm)	6.5 ± 0.4	6.3 ± 0.5	6.5 ± 0.4	6.5 ± 0.4	6.5 ± 0.3	6.3 ± 0.3	0.069	0.453	0.144
ICA diameter (mm)	5.0 ± 0.6	5.2 ± 0.8	4.9 ± 0.6	5.3 ± 0.9	5.3 ± 0.7	5.3 ± 0.9	0.151	0.112	0.151
CCA velocity (cm/s)	38 ± 5	42 ± 7*	37 ± 5	52 ± 8*	37 ± 5	46 ± 5*	<0.001	0.006	<0.001
ICA velocity (cm/s)	33 ± 7	40 ± 9*	36 ± 6	52 ± 8*	36 ± 6	45 ± 7*	<0.001	0.013	0.018
CCA shear rate (/s)	234 ± 42	268 ± 54*	232 ± 35	324 ± 57*	232 ± 39	292 ± 38*	<0.001	0.022	0.005
ICA shear rate (/s)	266 ± 67	314 ± 82*	298 ± 80	407 ± 107*	277 ± 77	356 ± 98*	<0.001	0.019	0.131

*Note*: Values are presented as means ± SD. *n* = 12 (7F/5M) for all variables except ICA diameter and shear rate (*n* = 11, 7F/4M), and blood viscosity (*n* = 9, 4F/5M). *P < 0.05 versus pre‐exercise. Abbreviations: CCA, common carotid artery; CO, cardiac output; DBP, diastolic blood pressure; HR, heart rate; ICA, internal carotid artery; MAP, mean arterial pressure; SBP, systolic blood pressure; SV, stroke volume; ${\dot{V}}_{O_{2}\max}$, maximal oxygen uptake.

### 3.1 CCA results

Under baseline conditions, mean CCA diameter did not differ across visits (Table 2; ICC = 0.8, *P *< 0.001). Following exercise, mean CCA diameter was not different from pre‐exercise and there were no differences across conditions (time: *P *= 0.069, condition: *P *= 0.453, interaction: *P *= 0.144). In contrast, mean CCA blood velocity was increased following exercise (Table 2; time: *P *< 0.001). However, this effect differed across conditions (condition: *P *= 0.006, interaction: *P *< 0.001). The increase in CCA blood velocity following exercise was greater in the 70% ${\dot{V}}_{O_{2}\max}$ condition (∆ 15 ± 5 cm/s) compared with the 30% ${\dot{V}}_{O_{2}\max}$ condition (∆4 ± 4 cm/s; *P *< 0.001, *d *= 1.8) but did not differ from the 70% ${\dot{V}}_{O_{2}\max}$ EqEE condition (∆ 9 ± 7 cm/s; *P *= 0.057, *d *= 0.8). Also, the increase in CCA blood velocity following the 70% ${\dot{V}}_{O_{2}\max}$ EqEE condition did not differ from the 30% ${\dot{V}}_{O_{2}\max}$ condition (*P *= 0.137, *d *= 0.7).

**FIGURE 1:**
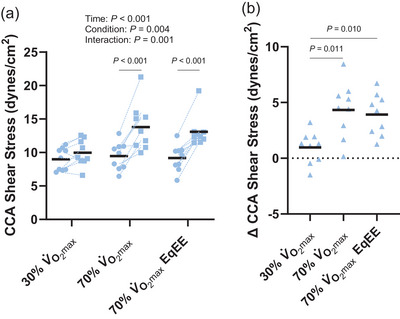
Common carotid artery (CCA) shear stress pre‐ (circles) and immediately post‐exercise (squares; a) and the change in CCA shear stress across conditions (b). Two‐way repeated measures analysis of variance with Tukey's *post hoc* analyses demonstrated that CCA shear stress was increased following exercise at 70% ${\dot{V}}_{O_{2}\max}$ and 70% ${\dot{V}}_{O_{2}\max}$ EqEE. The increase in CCA shear stress was greater following 70% ${\dot{V}}_{O_{2}\max}$ exercise and 70% ${\dot{V}}_{O_{2}\max}$ EqEE exercise compared with 30% ${\dot{V}}_{O_{2}\max}$ exercise. Mean (black line) and individual data (*n* = 9, 4F/5M) are shown.

**FIGURE 2:**
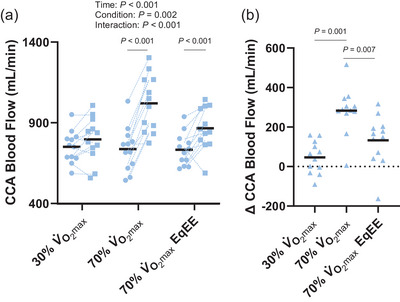
Common carotid artery (CCA) blood flow pre‐ (circles) and immediately post‐exercise (squares; a) and the change in CCA blood flow across conditions (b). Two‐way repeated measures analysis of variance with Tukey's *post hoc* analyses demonstrated that CCA blood flow was increased following exercise at 70% ${\dot{V}}_{O_{2}\max}$ and 70% ${\dot{V}}_{O_{2}\max}$ EqEE. The increase in CCA blood flow was greater following 70% ${\dot{V}}_{O_{2}\max}$ exercise than 70% ${\dot{V}}_{O_{2}\max}$ EqEE. Mean (black line) and individual data (*n* = 12, 7F/5M) are shown.

The effect of exercise on CCA shear stress differed by condition (Figure 1a; time: *P *< 0.001, condition: *P *= 0.004, interaction: *P *= 0.001) such that CCA shear stress was not different following exercise at 30% ${\dot{V}}_{O_{2}\max}$ (*P *= 0.154, *d *= 0.6) but was increased following the 70% ${\dot{V}}_{O_{2}\max}$ (*P *< 0.001, *d *= 1.7) and 70% ${\dot{V}}_{O_{2}\max}$ EqEE (*P *< 0.001, *d *= 2.2) conditions. However, the increase in CCA shear stress following exercise did not differ between exercise at 70% ${\dot{V}}_{O_{2}\max}$ (∆ 4.3 ± 2.5 dyn/cm^2^) compared with exercise at 70% ${\dot{V}}_{O_{2}\max}$ EqEE (Figure 1b; ∆ 3.9 ± 1.9 dyn/cm^2^, *P *= 0.846, *d *= 0.2). Delta CCA shear stress following 30% ${\dot{V}}_{O_{2}\max}$ exercise (∆ 1.0 ± 1.4 dyn/cm^2^) differed from 70% ${\dot{V}}_{O_{2}\max}$ exercise (*P *= 0.011, *d *= 1.3) and 70% ${\dot{V}}_{O_{2}\max}$ EqEE exercise (*P *= 0.010, *d *= 1.3). The change in CCA blood flow following exercise differed across conditions (Figure 2a; time: *P *< 0.001, condition: *P *= 0.002, interaction: *P *< 0.001). CCA blood flow was not increased following exercise at 30% ${\dot{V}}_{O_{2}\max}$ (*P *= 0.156, *d *= 0.6) but increased following the 70% ${\dot{V}}_{O_{2}\max}$ condition (*P *< 0.001, *d *= 2.2) and the 70% ${\dot{V}}_{O_{2}\max}$ EqEE condition (*P *< 0.001, *d *= 1.0). Following exercise at 70% ${\dot{V}}_{O_{2}\max}$, CCA blood flow increased 283 ± 119 mL/min, which was greater than the increase following exercise at 70% ${\dot{V}}_{O_{2}\max}$ EqEE (133 ± 126 mL/min, *P *= 0.007, *d *= 1.1; Figure 2b).

### 3.2 ICA results

Under baseline conditions, mean ICA diameter did not differ across conditions (Table 2; ICC = 0.8, *P *< 0.001). Mean ICA diameter was not different from pre‐exercise values following exercise in all conditions (time: *P *= 0.151, condition: *P *= 0.112, interaction: *P *= 0.151). Mean ICA blood velocity was increased with exercise (Table 2; time: *P *< 0.001) but the magnitude of effect differed across conditions (condition: *P *= 0.013, interaction: *P *= 0.018). The increase in ICA blood velocity was larger following exercise at 70% ${\dot{V}}_{O_{2}\max}$ (∆ 16 ± 8 cm/s) compared with exercise at 30% ${\dot{V}}_{O_{2}\max}$ (∆ 7 ± 5 cm/s; *P *= 0.016, *d *= 1.1). ICA blood velocity increased by 10 ± 9 cm/s following the 70% ${\dot{V}}_{O_{2}\max}$ EqEE condition, which did not differ from the 30% ${\dot{V}}_{O_{2}\max}$ (*P *= 0.592, *d *= 0.4) or the 70% ${\dot{V}}_{O_{2}\max}$ (*P *= 0.173, *d *= 0.6) condition.

**FIGURE 3:**
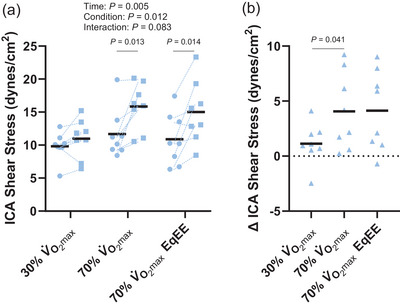
Internal carotid artery (ICA) shear stress pre‐ (circles) and immediately post‐exercise (squares; a) and the change in ICA shear stress across conditions (b). Two‐way repeated measures analysis of variance with Tukey's *post hoc* analyses demonstrated that ICA shear stress was increased following exercise at 70% ${\dot{V}}_{O_{2}\max}$ and 70% ${\dot{V}}_{O_{2}\max}$ EqEE. The increase in ICA shear stress was greater following 70% ${\dot{V}}_{O_{2}\max}$ exercise than 30% ${\dot{V}}_{O_{2}\max}$ exercise. Mean (black line) and individual data (*n* = 8, 4F/4M) are shown.

**FIGURE 4:**
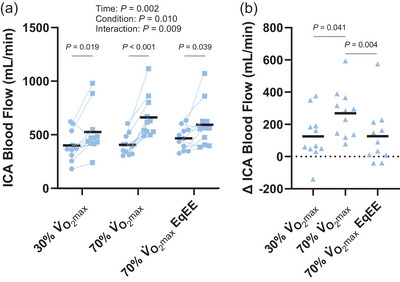
Internal carotid artery (ICA) blood flow pre‐ (circles) and immediately post‐exercise (squares; a) and the change in ICA blood flow across conditions (b). Two‐way repeated measures analysis of variance with Tukey's *post hoc* analyses demonstrated that ICA blood flow was increased following exercise at 30% ${\dot{V}}_{O_{2}\max}$, 70% ${\dot{V}}_{O_{2}\max}$ and 70% ${\dot{V}}_{O_{2}\max}$ EqEE. The increase in ICA blood flow was greater following 70% ${\dot{V}}_{O_{2}\max}$ exercise than 30% ${\dot{V}}_{O_{2}\max}$ and 70% ${\dot{V}}_{O_{2}\max}$ EqEE. Mean (black line) and individual data (*n* = 11, 7F/4M) are shown.

Exercise increased ICA shear stress (time: *P *= 0.005) and there was a main effect of condition (condition: *P *= 0.012, interaction: *P *= 0.083; Figure 3a). ICA shear stress was not different following exercise at 30% ${\dot{V}}_{O_{2}\max}$ (*P *= 0.128, *d *= 0.5) but was increased following the 70% ${\dot{V}}_{O_{2}\max}$ (*P *= 0.013, *d *= 1.1) and 70% ${\dot{V}}_{O_{2}\max}$ EqEE (*P *= 0.014, *d *= 1.1) conditions. However, the increase in shear stress following 70% ${\dot{V}}_{O_{2}\max}$ exercise (∆ 4.1 ± 3.5 dyn/cm^2^) did not differ from the increase following 70% ${\dot{V}}_{O_{2}\max}$ EqEE exercise (∆ 4.1 ± 3.6 dyn/cm^2^, *P *= 0.999, *d *= 0.0; Figure 3b). Further, the increase in ICA shear stress was greater following exercise at 70% ${\dot{V}}_{O_{2}\max}$ compared with the change in ICA shear stress following exercise at 30% ${\dot{V}}_{O_{2}\max}$ (∆ 1.1 ± 1.9 dyn/cm^2^; *P *= 0.041, *d *= 1.1). In contrast, the change in ICA shear stress following exercise was not different between the 30% ${\dot{V}}_{O_{2}\max}$ and the 70% ${\dot{V}}_{O_{2}\max}$ EqEE conditions (*P *= 0.174, *d *= 0.7). ICA blood flow increased following exercise (time: *P *= 0.002) and the effect differed by condition (condition: *P *= 0.010, interaction *P *= 0.009; Figure 4a). ICA blood flow increased 268 ± 150 mL/min following the 70% ${\dot{V}}_{O_{2}\max}$ condition, which was greater than the 30% ${\dot{V}}_{O_{2}\max}$ condition (∆ 125 ± 149 mL/min; *P *= 0.041, *d *= 0.8) and the 70% ${\dot{V}}_{O_{2}\max}$ EqEE condition (∆ 127 ± 177 mL/min; *P *= 0.004, *d *= 1.3; Figure 4b). However, the increase in ICA blood flow following exercise did not differ between exercise at 30% ${\dot{V}}_{O_{2}\max}$ and exercise at 70% ${\dot{V}}_{O_{2}\max}$ EqEE (*P *= 0.974, *d *= 0.01).

## 4 DISCUSSION

The present study provides novel insight into the independent and combined impact of exercise intensity and dose on cerebral shear stress and blood flow following acute exercise in healthy young adults. This study demonstrates that immediately following exercise, ICA shear stress was elevated with larger increases observed following exercise at 70% ${\dot{V}}_{O_{2}\max}$ compared with exercise at 30% ${\dot{V}}_{O_{2}\max}$. ICA blood flow was also elevated following exercise and larger increases occurred after 70% ${\dot{V}}_{O_{2}\max}$ exercise when compared with 30% ${\dot{V}}_{O_{2}\max}$ exercise. Despite similar increases in ICA shear stress following 70% ${\dot{V}}_{O_{2}\max}$ exercise and 70% ${\dot{V}}_{O_{2}\max}$ EqEE exercise, ICA blood flow increased to a greater extent following exercise at 70% ${\dot{V}}_{O_{2}\max}$ compared with exercise at 70% ${\dot{V}}_{O_{2}\max}$ EqEE. Therefore, vigorous intensity exercise performed at a higher dose (i.e. duration of 30 min vs. ∼14 min) produced larger increases in ICA blood flow following exercise despite similar increases in ICA shear stress. As such, both exercise intensity and dose are important determinants of the post‐exercise ICA shear stress and blood flow responses to acute exercise in healthy young adults, as higher intensity exercise when performed at a higher dose evoked the largest post‐exercise increase in ICA blood flow, which may benefit cerebrovascular function.

During graded dynamic cycling exercise, ICA blood flow increases up to approximately 60% ${\dot{V}}_{O_{2}\mathrm{peak}}$ before appearing to plateau and beginning to decrease at 80% ${\dot{V}}_{O_{2}\mathrm{peak}}$ (Sato & Sadamoto, 2010; Sato et al., 2011). In contrast, CCA blood flow continues to increase through 80% ${\dot{V}}_{O_{2}\mathrm{peak}}$ (Sato et al., 2011). Shear‐mediated dilatation represents an important mechanism contributing to the regulation of cerebral blood flow during exercise (Smith et al., 2019). Submaximal aerobic cycling and treadmill exercise at low‐, moderate‐ and high‐intensities produced graded elevations in CCA shear stress (Montalvo et al., 2022). In a study by Ogoh et al., dose‐matched continuous aerobic cycling exercise (80 W) and interval cycling exercise (average 80 W) produced elevations in ICA shear rate which remained increased during the initial phases of recovery (Ogoh et al., 2021). Despite greater elevations in ICA shear rate during interval exercise compared with continuous exercise, the increase in ICA blood flow was not different between exercise conditions (Ogoh et al., 2021). In the study by Ogoh et al. (2021), the average intensity of the continuous and interval exercise was similar, and by design the conditions were dose‐matched, which limited the ability to compare exercise intensity and dose effects on ICA shear and blood flow.

Variations in exercise intensity represent an important determinant of the shear patterns during exercise. To address the impact of exercise intensity on ICA shear stress and blood flow in the present study, participants performed treadmill exercise for 30 min at 30% ${\dot{V}}_{O_{2}\max}$ and at 70% ${\dot{V}}_{O_{2}\max}$. Specifically, these intensities were selected because (1) the pace to elicit 30% ${\dot{V}}_{O_{2}\max}$ was equivalent to a brisk walk capturing the intensity that many individuals exercise at on a regular basis, and (2) exercise at 70% ${\dot{V}}_{O_{2}\max}$ corresponds with previously reported peak cerebral blood flow during exercise (Sato & Sadamoto, 2010; Sato et al., 2011). In the present study, we demonstrated differences in ICA shear stress and blood flow following 30% ${\dot{V}}_{O_{2}\max}$ and 70% ${\dot{V}}_{O_{2}\max}$ exercise. We report that immediately following treadmill exercise, ICA shear stress and blood flow were greater after 70% ${\dot{V}}_{O_{2}\max}$ exercise compared with 30% ${\dot{V}}_{O_{2}\max}$ exercise and similar patterns occurred in the CCA. Previously, Sakamoto et al. (2021) compared ICA shear rate and mean blood flow responses during cycling exercise between moderate‐intensity exercise (∼65% HR_max_) and high‐intensity exercise (∼85% HR_max_) performed for 30 min. In contrast to the present study, ICA shear rate remained unchanged during exercise and was not different between exercise intensities. Further, despite ICA blood flow not changing from rest, there was a trend for a decrease in ICA flow during high‐intensity exercise. As such, ICA blood flow was different between exercise intensities with lower blood flow observed during high intensity exercise relative to moderate intensity exercise (Sakamoto et al., 2021). The trend for decreases in ICA blood flow during the high‐intensity exercise in the previous investigation (Sakamoto et al., 2021) are in line with previous reports of decreasing ICA blood flow observed at 80% ${\dot{V}}_{O_{2}\mathrm{peak}}$, which was not different from resting values (Sato et al., 2011). In line with this point, the variations in the high‐intensity exercise conditions may explain observed differences between the present study and the investigation by Sakamoto et al. (2021). Indeed, ICA blood flow plateaus around 70% ${\dot{V}}_{O_{2}\mathrm{peak}}$ (Sato & Sadamoto, 2010) which is similar to the vigorous intensity exercise performed in the present study. Further, it is unclear why ICA shear rate was unchanged during moderate‐ and high‐intensity exercise in the investigation by Sakamoto et al. (2021), but the lack of changes in $ET_{CO_{2}}$ during the moderate‐intensity exercise, the fitness level of participants, and body posture during exercise may have influenced the differing results between the two studies. The present findings of increased ICA and CCA shear and blood flow following exercise are in line with existing studies investigating ICA responses (Ogoh et al., 2021; Sato & Sadamoto, 2010; Sato et al., 2011) and CCA responses (Hellstrom et al., 1996; Montalvo et al., 2022; Sato et al., 2011) to exercise.

While vigorous intensity exercise produced greater elevations in ICA shear stress and blood flow following exercise, the larger dose of exercise (greater energy expenditure) obtained with the 70% ${\dot{V}}_{O_{2}\max}$ exercise compared with the duration‐matched 30% ${\dot{V}}_{O_{2}\max}$ represents an important consideration. The present study further explored the post‐exercise effect of exercise intensity by comparing dose‐equivalent treadmill exercise (matched energy expenditure) performed at 30% and 70% ${\dot{V}}_{O_{2}\max}$. While dose‐equivalent exercise (30% ${\dot{V}}_{O_{2}\max}$ and 70% ${\dot{V}}_{O_{2}\max}$ EqEE) elicited differences in post‐exercise CCA shear stress, no differences were observed between dose‐equivalent conditions in post‐exercise ICA shear stress or blood flow. These findings suggest that both exercise intensity and dose are important considerations for ICA shear and blood flow responses following acute exercise. Indeed, vigorous intensity exercise (70% ${\dot{V}}_{O_{2}\max}$) performed for varying durations (30 min vs. 14 min) produced differences in post‐exercise ICA blood flow responses. Specifically, despite similar increases in CCA and ICA shear stress between the intensity‐matched exercise conditions, a longer exercise duration, at the same intensity, produced greater increases in post‐exercise CCA and ICA blood flow. To the best of our knowledge, this study is the first to quantify the effect of exercise dose on acute post‐exercise ICA shear stress and blood flow. A previous investigation in the peripheral circulation found no differences in post‐exercise brachial artery shear rate between 50% ${\dot{V}}_{O_{2}\max}$ for 30 min and 50% ${\dot{V}}_{O_{2}\max}$ for 60 min (Johnson & Wallace, 2012). The contrasting findings between the peripheral and cerebral circulation are not unexpected, as differing peripheral and cerebral shear responses to exercise of varying intensity have been reported (Amin et al., 2022).

Endothelial shear‐mediated mechanisms represent a key regulator of blood flow responses during exercise (Niebauer & Cooke, 1996). While much of the existing literature in the area has relied on shear rate using only measures of blood velocity and diameter, the present study reports shear stress determined from measures of blood viscosity, blood velocity, and diameter. Measures of shear stress are critical for post‐exercise measures of arterial shear as exercise of varying intensities and dose differentially affected the change in blood viscosity pre‐ to post‐exercise. Indeed, using measures of shear rate fails to detect a larger increase in CCA shear following 70% ${\dot{V}}_{O_{2}\max}$ exercise than 70% ${\dot{V}}_{O_{2}\max}$ EqEE exercise, which is observed with measures of shear stress. Shear stress in the ICA remained elevated following exercise in all conditions and 70% ${\dot{V}}_{O_{2}\max}$ exercise provoked larger increases when compared to 30% ${\dot{V}}_{O_{2}\max}$ exercise, a difference that measures of ICA shear rate did not detect. In line with the larger elevations in ICA shear stress following 70% ${\dot{V}}_{O_{2}\max}$ exercise, ICA blood flow also increased to a greater extent compared to the 30% ${\dot{V}}_{O_{2}\max}$ condition. In contrast, despite similar increases in ICA shear stress following 70% ${\dot{V}}_{O_{2}\max}$ exercise and 70% ${\dot{V}}_{O_{2}\max}$ EqEE exercise, ICA blood flow increased to a greater extent following 70% ${\dot{V}}_{O_{2}\max}$ exercise and similar patterns occurred in the CCA. While post‐exercise shear stress was not different between the two conditions at the time of measurement, we speculate that 70% ${\dot{V}}_{O_{2}\max}$ (30 min) would produce a greater shear stimulus area under the curve during exercise when compared with 70% ${\dot{V}}_{O_{2}\max}$ EqEE (∼14 min). The greater shear stimulus area under the curve would produce greater downstream dilatation (Hoiland et al., 2022, 2017; Pyke & Tschakovsky, 2007) and subsequently greater increases in blood flow, and future studies could address this idea. In contrast to the role of shear‐mediated mechanisms, blood pressure is not expected to have contributed significantly to the differences in post‐exercise CCA and ICA blood flow responses between exercise conditions as MAP following exercise was not different from pre‐exercise values and no differences were observed across conditions. Variations in $ET_{CO_{2}}$ following exercise is another factor that could contribute to differences in blood flow. However, $ET_{CO_{2}}$ was 1–2 mmHg lower, at 39–40 mmHg post‐exercise and the reductions were similar between conditions.

### 4.1 Methodological considerations

There are methodological considerations for the present study. First, in the present study we focused on the post‐exercise period to capture ICA haemodynamics. To determine the impact of acute exercise, and the influence of exercise intensity and dose, on post‐exercise cerebrovascular regulation, it is important to understand variations in post‐exercise cerebral shear stress, cerebral blood flow and central haemodynamics, which may act as a stimulus for upregulation of cerebrovascular regulatory mechanisms. Previous studies in the cerebral circulation demonstrated that the effects of exercise intensity on ICA shear rate, MCAv and ICA blood flow persisted following exercise (Ogoh et al., 2005, 2021; Sato & Sadamoto, 2010). However, the time frame in which cerebral blood flow and shear stress remained elevated post‐exercise remains unknown, as only one time point was evaluated immediately post‐exercise in the present study (roughly 3–6 min post‐exercise). Second, the sample in the present study was healthy, physically active young adults. With an average relative ${\dot{V}}_{O_{2}\max}$ of 53 ± 9 mL/kg/min, the participants in the present study were aerobically trained. This was intentional in the study design to understand the impact of exercise intensity and dose in young aerobically trained adults before expanding to other populations. Nevertheless, the influence of acute exercise intensity and dose on ICA shear stress and blood flow may differ in less active or more sedentary populations as well as in middle‐aged and older adults. Third, biological sex may affect the ICA shear stress and blood flow responses to acute exercise of varying intensity and dose. The small sample size in the present study limited assessments of sex differences in ICA responses to acute exercise. Importantly, females completed study visits during days 2–6 of their menstrual cycle when levels of ovarian sex hormones are low to limit the potential variability in ICA shear stress and blood flow responses to exercise that may stem from the menstrual cycle. Nonetheless, future research with a larger sample size should aim to understand the impact of both biological sex and the menstrual cycle on acute ICA shear stress and blood flow responses to exercise. Fourth, increases in core body temperature during aerobic exercise differentially mediate increases in blood flow in the extracranial arteries. Specifically, passive heat stress increased external carotid artery (ECA) and vertebral artery blood flow to a similar extent as exercise whereas ICA blood flow was unchanged during passive heat stress (Caldwell et al., 2020). Additionally, exercise with heat stress elevated CCA and ECA blood flow to a greater extent than exercise without heat stress while no differences were observed between conditions in ICA blood flow (Sato et al., 2016). While it is unknown whether these effects persist following aerobic exercise, differences in exercise‐mediated heat stress between conditions in the present study could have influenced CCA responses following exercise but are not expected to have affected ICA responses following exercise. Furthermore, all study visits were performed in a temperature‐controlled room to minimize excessive increases in core body temperature. Fifth, this study did not examine dose‐dependent effects of light intensity exercise (e.g., 30% ${\dot{V}}_{O_{2}\max}$). Therefore, the dose‐dependent effects of exercise that were observed with vigorous intensity exercise cannot be generalized to exercise performed at a lighter intensity such as 30% ${\dot{V}}_{O_{2}\max}$.

### 4.2 Conclusions

This study reports novel findings regarding the independent and combined effects of exercise intensity and dose on acute post‐exercise cerebral shear stress and blood flow. Our results suggest that exercise intensity did not impact post‐exercise ICA blood flow when energy expenditure matched exercise was performed. In contrast, vigorous intensity exercise at a higher dose (running at 70% ${\dot{V}}_{O_{2}\max}$ for 30 min) produced the largest increases in ICA blood flow following exercise compared with lower intensity exercise performed for 30 min (30% ${\dot{V}}_{O_{2}\max}$) and vigorous intensity exercise at a lower dose (70% ${\dot{V}}_{O_{2}\max}$ for 14 min). Therefore, exercise intensity and overall dose are important considerations when examining post‐exercise cerebral haemodynamics and cerebrovascular function. These findings may inform future work aimed at utilizing aerobic exercise as a strategy for acutely improving cerebrovascular function.

## AUTHOR CONTRIBUTIONS

Adam T. Corkery, Anna J. Howery, Jill N. Barnes conceived and designed research; Adam T. Corkery, Kathleen B. Miller, Andrew G. Pearson, Nicole A. Loggie, Anna J. Howery, Jill N. Barnes performed experiments; M. Erin Moir, Adam T. Corkery, Avery A. Apfelbeck analysed data; M. Erin Moir, Adam T. Corkery, Kathleen B. Miller, Andrew G. Pearson, Nicole A. Loggie, Anna J. Howery, Jill N. Barnes interpreted results; M. Erin Moir, Adam T. Corkery, Jill N. Barnes drafted the manuscript; M. Erin Moir, Adam T. Corkery, Kathleen B. Miller, Andrew G. Pearson, Nicole A. Loggie, Avery A. Apfelbeck, Anna J. Howery, Jill N. Barnes edited and revised the manuscript. All authors have read and approved the final version of this manuscript and agree to be accountable for all aspects of the work in ensuring that questions related to the accuracy or integrity of any part of the work are appropriately investigated and resolved. All persons designated as authors qualify for authorship, and all those who qualify for authorship are listed.

## CONFLICT OF INTEREST

None.

## Data Availability

The data are available upon reasonable request to the corresponding author.
